# Suicide mortality and suicidal ideation among patients with colorectal cancer: a systematic review and meta-analysis

**DOI:** 10.1016/j.eclinm.2025.103670

**Published:** 2025-11-28

**Authors:** Sebastian Bogner, Corinna Seliger-Behme, Michael F. Leitzmann, Patricia Bohmann

**Affiliations:** a Department of Epidemiology and Preventive Medicine, University of Regensburg, Regensburg, Germany; b Department of Neurology, University Hospital Knappschaftskrankenhaus Bochum, Ruhr University Bochum, Bochum, Germany; c Department of Neurology, Medbo District Hospital and University Hospital Regensburg, Regensburg, Germany

**Keywords:** Suicide, Suicidal ideation, Colorectal cancer, Meta-analysis

## Abstract

**Background:**

Receiving a cancer diagnosis is strongly associated with a higher risk of suicide. However, studies examining suicidality in patients with colorectal cancer show some inconsistencies, particularly concerning factors such as disease stage or specific diagnosis.

**Methods:**

We conducted a systematic review and meta-analysis to investigate the association between colorectal cancer and suicide or suicidal ideation. EMBASE, MEDLINE, PsycINFO, Science Citation Index Expanded & Social Sciences Citation Index, CINAHL, and Google Scholar were searched from database inception to May 31, 2025. Eligible studies included longitudinal cohort or case–control designs involving patients with colorectal cancer aged ≥14 years. Control groups comprised individuals from the same population without cancer. Data were independently extracted by two researchers from published reports available in English or German. The primary outcome was suicide, defined as death from intentional self-harm; the secondary outcome was suicidal ideation, defined as non-fatal thoughts of suicide. We performed random-effects meta-analyses, assessing heterogeneity with Q and I^2^ statistics and publication bias with funnel plots, Begg's, and Egger's tests. The study was registered (PROSPERO: CRD420251051277).

**Findings:**

Among 4,700 records screened, 44 studies met the inclusion criteria, encompassing at least 9,385,472 patients with colorectal cancer and 13,308 suicides. Of these, 34 studies reported Standardised Mortality Ratios (SMR; colorectal cancer patients: n = 8,251,924; suicides: n = 12,081) and were included in the meta-analysis. After excluding studies with potential overlap in patient populations, the primary analysis was based on nine independent studies including at least 1,204,072 individuals with colorectal cancer, of whom 2,731 died by suicide. For suicidal ideation, we report the results of five individual studies. All included studies met methodological quality criteria, with a Newcastle–Ottawa Scale score of ≥7. The findings indicate a significantly increased suicide risk for patients with colorectal cancer, with a pooled SMR of 1.40 (95% CI: 1.33–1.49, I^2^ = 28.17%, no evidence for publication bias) compared to the general population. Subgroup analyses revealed notably higher suicide risks among patients with metastatic disease (SMR = 3.63, 95% CI: 2.99–4.41), those under 40 years of age (SMR = 2.15, 95% CI: 1.60–2.88), and individuals diagnosed within the past six months (SMR = 2.69, 95% CI: 1.29–5.61). For suicidal ideation, primary studies did not observe differences between patients with colorectal cancer and their reference groups, such as cancer-free individuals (SMR = 1.70, 95% CI: 0.65–4.42) or patients with hepatic cancer (SMR = 1.14, 95% CI: 0.94–1.38).

**Interpretation:**

Our results indicate the need for comprehensive psychological screening in patients with colorectal cancer, who show a substantially higher suicide risk than the general population. Particular attention should be given to vulnerable subgroups, including those with metastatic disease, younger patients, and those recently diagnosed. Implementing these results into clinical practice can help facilitate patient-centred, cost-effective psycho-oncological care. Notably, evidence from low- and middle-income countries remains scarce, and younger populations might be underrepresented, indicating that our results should be interpreted with caution for these groups.

**Funding:**

None.

**Research in context**

**Evidence before this study**

Before undertaking this study, we searched PubMed, EMBASE, PsycINFO, Web of Science database, CINAHL, and Google Scholar to identify existing evidence on the incidence of suicide and suicidal ideation among patients with colorectal cancer. The search covered all records from database inception to October 15, 2024, with no restrictions on language or publication date. We used combinations of terms for three main concepts: (1) cancer, with an emphasis on “colorectal cancer”, “colon cancer” and “rectal cancer”; (2) suicide-related outcomes, including “suicide”, “suicidal ideation”, and “assisted death”; and (3) epidemiologic study designs, including “risk”, “cohort”, “population-based”, “observational”. These terms were combined with Boolean operators (e.g., “colorectal cancer” AND “suicide”) and expanded with database-specific subject headings (e.g., MeSH) and keywords. We identified several individual observational studies reporting suicide or suicidal ideation among patients with colorectal cancer. We also found one broader review of suicide in patients with cancer, published in 2022, which reported that individuals with colon and rectal cancer had an elevated suicide risk based on data pooled from 10 studies; however, colorectal cancer was not the primary focus of the review, and no subgroup analyses were presented. The quality of the available colorectal cancer studies varied, with common limitations including lack of adjustment for stage and treatment. No systematic review or meta-analysis focused specifically on suicidality in colorectal cancer was identified.

**Added value of this study**

Our meta-analysis of over 1.2 million patients with colorectal cancer found that suicide risk was significantly elevated compared to the general population, especially among patients with metastatic disease, diagnosed within six months, or under 40 years. No significant differences in suicidal ideation were found between patients with colorectal cancer and reference groups.

**Implications of all the available evidence**

Our findings suggest that colorectal cancer may be associated with elevated suicide risk, especially among metastatic, recently diagnosed, or younger patients. These findings emphasise the importance of comprehensive psychological screening and support in this population. However, at the same time, suicidal ideation did not differ between patients with colorectal cancer and respective reference groups. This discrepancy may reflect limitations in current assessment methods and highlights the importance of refining screening tools.

## Introduction

Between 1990 and 2021, the global suicide rate declined by 39.5%.^1^ Nevertheless, suicide remains a tragic yet often preventable cause of death, accounting for more than one in 100 deaths in 2019.^2^ Suicide risk is influenced by several factors, including psychiatric diseases, prior suicide attempts, and stressful life events.^1^^,^^2^ A cancer diagnosis is one such event and often causes significant emotional distress.^3^ Patients face pain, uncertainty, disruptions in social roles, and fear of premature death.^4^ These burdens can trigger suicidal ideation or suicide. We previously reported an 85% higher suicide mortality among cancer patients compared to the general population.^5^

Colorectal cancer, the third most common cancer worldwide,^6^ accounts for approximately 10% of all cancer diagnoses. Patients with colorectal cancer frequently experience fatigue, abdominal pain, and weight loss, impairing daily functioning and quality of life.^7^ Many report difficulty maintaining everyday activities like shopping, exercise, and social engagement,^8^ often causing psychological distress, anxiety, or depression.^9^ If unrecognised and untreated, such deterioration may lead to suicidal ideation or suicide attempts.

Several studies found that individuals with colorectal cancer show an increased risk of suicide,^10^, ^11^, ^12^, ^13^, ^14^, ^15^ though findings vary across subgroups. Some suggest higher risk shortly after diagnosis,^14^^,^^16^^,^^17^ while others highlight advanced-stage disease.^14^^,^^15^^,^^18^ Findings also vary by sex, with some studies reporting higher risk in males,^19^, ^20^, ^21^ and others in females.^14^^,^^22^^,^^23^

These inconsistencies, together with the high global incidence, its projected outpaced growth by 2070,^24^ and its wide range of sequelae, highlight the need for a colorectal cancer-specific systematic synthesis. Although numerous studies have examined suicide risk^10^, ^11^, ^12^, ^13^, ^14^, ^15^, ^16^, ^17^, ^18^, ^19^, ^20^, ^21^, ^22^, ^23^ and suicidal ideation^25^, ^26^, ^27^, ^28^, ^29^ in patients with colorectal cancer, no specific meta-analysis exists. Therefore, this study presents a systematic review and meta-analysis on the risk of suicide and suicidal ideation in this population.

## Methods

### Search strategy and selection criteria

We performed a systematic literature review and meta-analysis of studies published in English or German involving patients aged 14 years or older. Eligible studies were longitudinal cohort (retrospective or prospective) or case–control designs based on population or cancer registry data. Editorials, abstracts, and letters were excluded.

We searched MEDLINE (PubMed, from 1946), EMBASE (Ovid, from 1974), PsycINFO (EBSCOhost, from 1884), Science Citation Index Expanded and Social Sciences Citation Index (Web of Science from 1965 to 1990, respectively), CINAHL (EBSCOhost, from 1981), and Google Scholar from inception to May 31, 2025. We also screened the reference lists of included studies. Full search terms are reported in Supplementary Table S1. Two authors (SB, PB) independently screened titles, abstracts, and full texts; a third researcher (ML) resolved disagreements. This study was registered on PROSPERO (ID: CRD420251051277) and conducted following the PRISMA,^30^ MOOSE,^31^ and PRESS^32^ guidelines.

### Data analyses

Our analyses focused on patients with colorectal cancer, defined per study and harmonised to ICD-10 codes C18-20 (Supplementary Table S2)^33^; studies using earlier ICD versions or other classifications were included after confirming comparability with our ICD-10-based case definition.

The primary outcome was suicide, defined as death by intentional self-harm (harmonised to ICD-10: X60-X84, Y87.0).^34^ Studies reporting deaths of undetermined or unclassifiable causes were excluded. Risk was assessed using Standardised Mortality Ratios (SMR), Hazard Ratios (HR), Odds Ratios (OR), or Relative Risks (RR). Studies without these risk estimates (RE) or without corresponding 95% confidence intervals (CIs) were excluded.

The secondary outcome was suicidal ideation, defined as non-fatal thoughts of suicide, coded as ICD-10 R45.8 and measured by OR.^35^ As mentioned above, studies employed various classification systems depending on recruitment period and study region; to ensure comparability across studies, we converted all codes to our ICD-10-based definitions of the exposure and outcomes. Control groups comprised individuals from the same population without cancer.

All records were compiled and de-duplicated (n = 1,933) using Rayyan.^36^ Data were extracted by two authors (SB, PB) and subsequently verified for accuracy. The following information was extracted from each study: first author, title, journal, publication year, PMID/DOI, country, study design, database source, start of recruitment, outcome (suicide/suicidal ideation), inclusion/exclusion criteria, follow-up time, diagnosis, gender, race/ethnicity, age at diagnosis, extent of disease, therapy, time since diagnosis, control group, observed/expected numbers of suicide, type and value of RE, 95% CI, and adjustment factors. If several models were reported, the most comprehensively adjusted model was used. Study quality was assessed by two researchers (SB, PB) using the Newcastle–Ottawa Scale (NOS)^37^ (Supplementary Table S3). Only studies scoring seven or more points were classified as high quality and included in our analyses; those scoring less were excluded during full-text screening. The data was organised using Microsoft Excel (Version 2024).

For the main analysis, we focused on the most frequently reported RE–SMRs–to ensure greater homogeneity and comparability. Studies reporting HRs and ORs were analysed separately to complement the main analysis, while studies using RRs were excluded due to insufficient data. We pooled SMRs, HRs, and ORs in separate analyses to preserve interpretability and limit heterogeneity. These metrics estimate different quantities and rest on different denominators and assumptions; mixing them would inflate between-study heterogeneity and obscure clinical interpretation.

Only one study regarding suicidal ideation, our secondary outcome, used the pre-defined reference group,^29^ limiting us to a qualitative synthesis.

For suicide risk, all REs were meta-analysed using random-effects models.

For each analysis, we calculated the log-transformed RE and its standard error, s_i_ = d_i_/1.96, where d_i_ is the maximum of the distances between log(RE_i_) and its 95% CI bounds. Between-study variance (*τ*^2^) was calculated via restricted maximum-likelihood estimate.^38^ Individual study weights were defined as wᵢ = 1/(sᵢ^2^ + *τ*^2^), where sᵢ^2^ is the sampling variance of log(RE).

Study heterogeneity was assessed using Q and I^2^ statistics.^39^ Publication bias was evaluated using funnel plots for qualitative and Begg's^40^ and Egger's^41^ tests for quantitative assessment.

To avoid patient overlap, we excluded studies with potentially overlapping populations, such as those using the same databases or registries within overlapping time periods.^42^ A sensitivity analysis included all eligible studies, regardless of potential patient overlap, to maximise information.

Subgroup meta-regressions examined suicide risk factors in patients with colorectal cancer by gender, age, region, diagnosis, disease extent, time since diagnosis, and recruitment start. We used a Wald chi-square test to assess significance and applied false discovery rate correction for multiple testing, reporting results as q-values.^43^

All statistical analyses were conducted using the ‘metafor’ package in R (version 4.3.2).^38^

### Role of the funding source

There was no funding source for this study. SB and PB had full access to all study data and take responsibility for the integrity of the data and the accuracy of the data analysis. All authors confirmed the final version and agreed to submit this manuscript for publication.

## Results

### Study eligibility

**Fig. 1:**
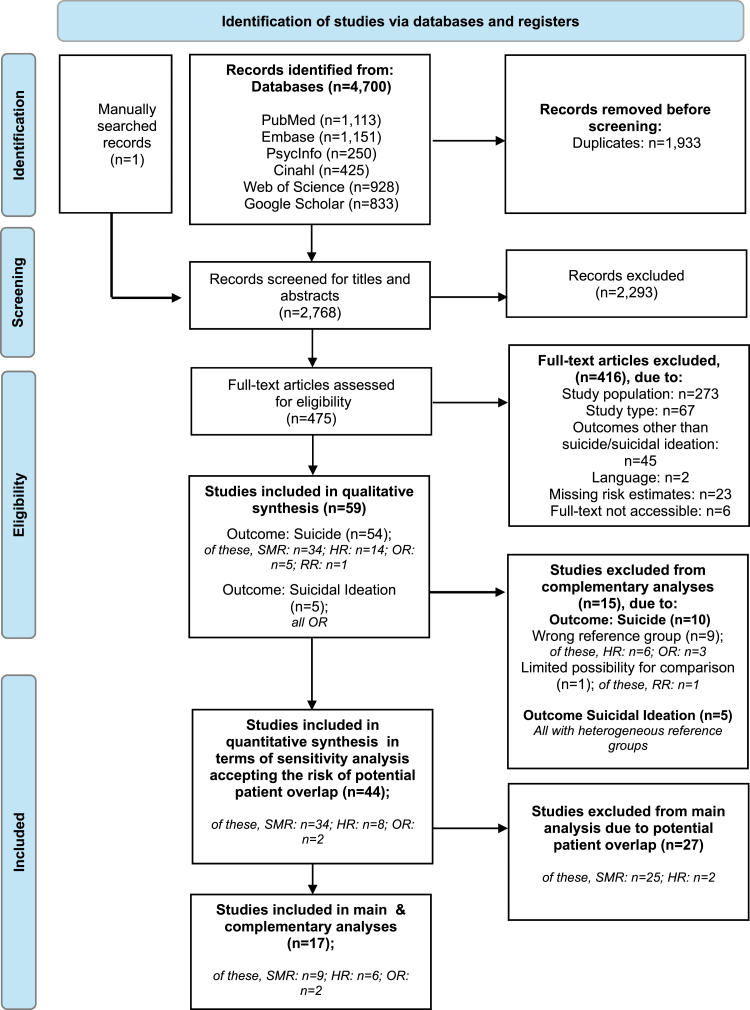
**PRISMA Flow Diagram for study selection**. PRISMA Flow Diagram illustrates the study selection process for the systematic review and meta-analysis. The diagram shows the number of records identified through database searching and other sources, the number of duplicates removed, records screened, full-text articles assessed for eligibility, and studies included in the final analysis. Boxes represent each step of the screening process, with ‘n=’ indicating the quantity of records at each stage. Exclusion reasons are specified for full-text articles.

The systematic literature search identified 4,700 records (PubMed: 1,113; EMBASE: 1,151; PsycINFO: 250; CINAHL: 425; Web of Science: 928; Google Scholar: 833) and one through manual search.^44^ After removing 1,933 duplicates, 2,768 articles were screened for title and abstract, of which 2,293 were excluded, leaving 475 studies for full-text screening. Of these, we excluded 273 articles for unsuitable study populations, 67 for study type, 45 for non-suicide outcomes, two for language, 23 due to lacking REs, and six without full texts. In total, 59 studies were eligible: 54 on suicide and five on suicidal ideation. The PRISMA flowchart shows the selection process and exclusion criteria (Fig. 1).

Regarding suicide, 34 studies provided SMRs,^13^, ^14^, ^15^, ^16^, ^17^, ^18^, ^19^, ^20^, ^21^, ^22^, ^23^^,^^44^, ^45^, ^46^, ^47^, ^48^, ^49^, ^50^, ^51^, ^52^, ^53^, ^54^, ^55^, ^56^, ^57^, ^58^, ^59^, ^60^, ^61^, ^62^, ^63^, ^64^, ^65^, ^66^ 14 HRs,^4^^,^^10^, ^11^, ^12^^,^^67^, ^68^, ^69^, ^70^, ^71^, ^72^, ^73^, ^74^, ^75^, ^76^ five ORs,^77^, ^78^, ^79^, ^80^, ^81^ and one RR.^82^ Since some studies drew data from the same cancer registries with overlapping recruitment periods, 25 studies reporting SMRs^13^^,^^15^^,^^18^, ^19^, ^20^, ^21^^,^^44^^,^^46^^,^^47^^,^^49^, ^50^, ^51^, ^52^^,^^54^, ^55^, ^56^, ^57^^,^^59^, ^60^, ^61^, ^62^, ^63^, ^64^, ^65^, ^66^ were excluded due to potential patient overlap, leaving nine independent studies^14^^,^^16^^,^^17^^,^^22^^,^^23^^,^^45^^,^^48^^,^^53^^,^^58^ for our main analysis. For complementary analyses, we excluded six HR^4^^,^^10^^,^^68^^,^^70^^,^^73^^,^^76^ and three OR studies^77^^,^^79^^,^^80^ that did not match our pre-defined control group. Of the remaining HR studies, two^69^^,^^74^ were excluded due to patient overlap. The two OR studies^78^^,^^81^ used different databases, posing no overlap risk. For detailed information see Supplementary Table S4.

### Main analysis: suicide risk

The nine SMR studies for our main analysis included at least 1,204,071 colorectal cancer patients with 2,731 suicides over 2,669,708 person-years. Across these studies, the number of patients with colorectal cancer ranged from 19,409 to 578,270. Three studies started recruitment before 1980. Two studies each were from Asia, Western Europe, Eastern Europe, and Scandinavia; one study was from the US. See Table 1 for details.

**Table 1:** Characteristics of studies meeting inclusion criteria for the main analysis.

Author, Year	Country	Time of Recruitment	Colorectal Cancer Patients	Suicides	Person-Years	Standardised mortality rate (95% CI)
Allebeck et al., 1989	Sweden	1975–1985	n.a.	133	n.a.	2.10 (1.60–3.20)
Hem et al., 2004	Norway	1960–1999	n.a.	63	318,934	1.14 (0.75–1.68)
Vyssoki et al., 2015	Austria	1983–2010	n.a.	396	1,851,586	1.46 (1.32–1.61)
Dulskas et al., 2019	Lithuania	1998–2012	19,409	67	85,505.6	1.62 (1.27–2.06)
Oh et al., 2019	South Korea	2000–2016	334,320	673	n.a.	1.50 (1.30–1.69)
Henson et al., 2019	England	1995–2015	578,270	349	n.a.	1.28 (1.15–1.42)
Liu et al., 2022	USA	1975–2016	n.a.	721	n.a.	1.34 (1.24–1.44)
Michalek et al., 2023	Poland	2009–2019	178,267	134	n.a.	1.46 (1.22–1.73)
Kitagawa et al., 2024	Japan	1985–2013	93,805	195	413,682	1.36 (1.09–1.70)

**Fig. 2:**
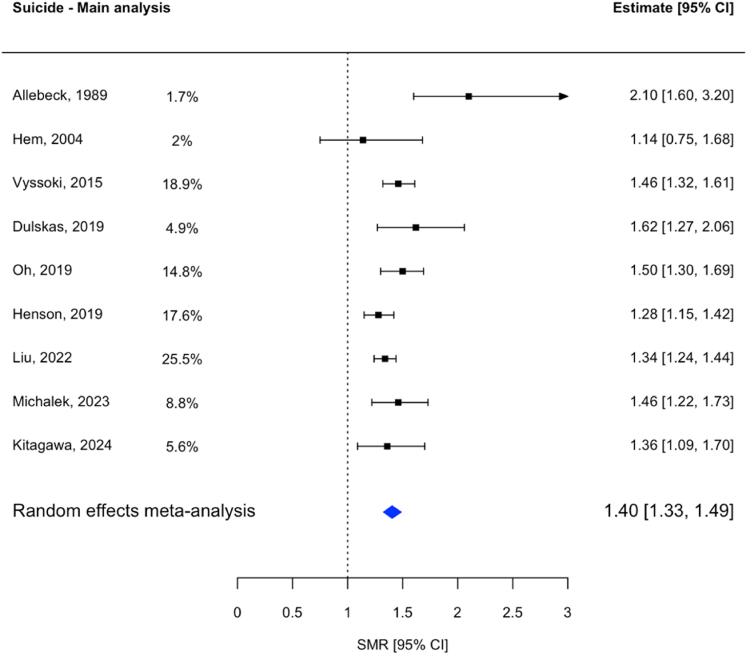
**Forest Plot of the results of the main analysis**. The Forest Plot shows the results of the nine individual studies included in the main analysis on suicide in colorectal cancer patients. The blue diamond shows the result of the random-effects meta-analysis, with the ends reflecting the confidence interval.

The pooled SMR of these nine studies comparing patients with colorectal cancer to the general population was 1.40 (95% CI: 1.33–1.49). While most studies reported a higher suicide risk, ranging from 1.28 (95% CI: 1.15–1.42)^17^ to 2.10 (95% CI: 1.60–3.20),^23^ one study did not show a statistically significant association (Fig. 2).^45^ Heterogeneity was moderate, with an I^2^ of 28.17%.

The sensitivity analysis, including all 34 eligible SMR studies regardless of potential patient overlap, confirmed this finding, with an SMR of 1.66 (95% CI: 1.52–1.82), including studies with SMRs ranging from 1.14 (95% CI: 0.75–1.68)^45^ to 3.38 (95% CI: 3.12–3.66; Supplementary Figure S1).^67^ This analysis showed higher heterogeneity (I^2^ = 93.99%).

Regarding the main analysis, the funnel plot suggested minimal asymmetry; however, both Begg's correlation (p = 0.36) and Egger's regression test (p = 0.21) indicated no evidence of publication bias or asymmetry. The Trim-and-Fill method identified one potentially missing study (Supplementary Figure S2A).

Regarding the sensitivity analysis, visual inspection of the funnel plot revealed minimal asymmetry. Nevertheless, Begg's (p = 0.20) and Egger's test (p = 0.19) did not indicate significant asymmetry. The Trim-and-Fill method suggested the presence of potentially missing studies on the left side of the funnel plot, hinting at a slight chance of publication bias (Supplementary Figure S2B).

### Main analysis: suicidal ideation

**Fig. 3:**
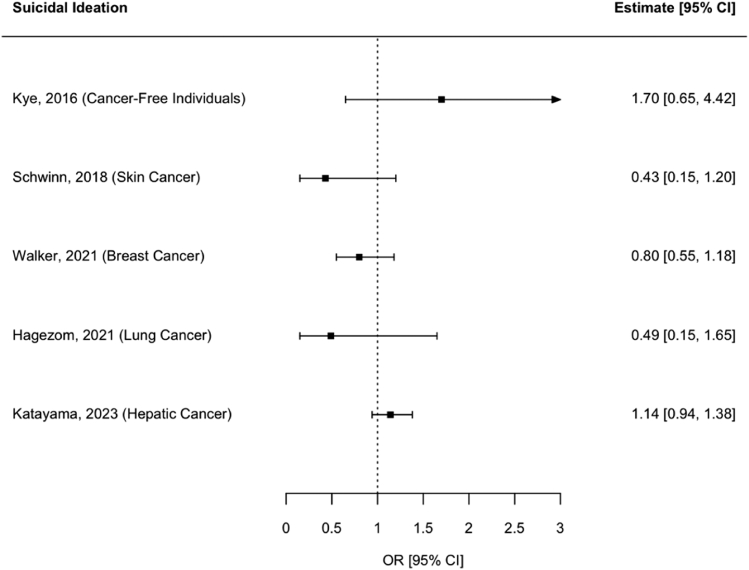
**Forest plot of studies on suicidal ideation**. The Forest Plot shows the results of the five individual studies on suicidal ideation in patients with colorectal cancer. The respective reference groups are given in brackets after publication year.

Regarding suicidal ideation, five studies provided ORs.^25^, ^26^, ^27^, ^28^, ^29^ Four studies reported ORs for suicidal ideation in colorectal cancer vs. various other cancer type patients, and only one study compared to cancer-free individuals, making meta-analysis unfeasible. None of these five studies^25^, ^26^, ^27^, ^28^, ^29^ found significant differences between patients with colorectal cancer and their respective reference group (Fig. 3). Kye et al.,^29^ the only study with a cancer-free control group, reported a non-significant increase in suicidal ideation risk (OR = 1.70, 95% CI: 0.65–4.42). The other four studies^25^, ^26^, ^27^, ^28^ compared patients with colorectal cancer to other cancer types, with ORs ranging from 0.43 (95% CI: 0.15–1.20; comparison with skin cancer)^25^ to 1.14 (95% CI: 0.94–1.38; comparison with hepatic cancer).^26^

### Subgroup analyses

**Fig. 4:**
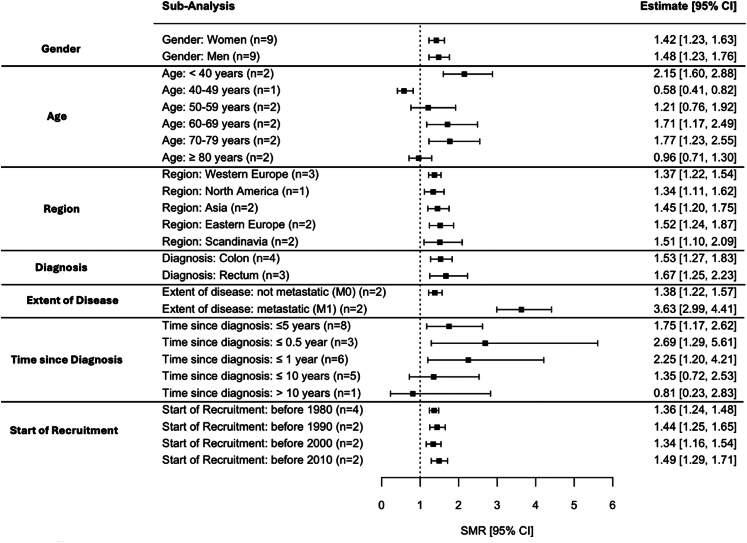
**Summary forest plot of subgroup analyses**. The Forest Plot shows the results of the seven subgroup analyses, including the nine non-overlapping studies. In each category, the first group listed serves as the reference group. ‘n=’ indicates the number of studies which provided data on this respective subgroup.

Gender-specific analysis showed comparable SMRs for women (1.42, 95% CI: 1.23–1.63) and men (1.48, 95% CI: 1.23–1.76). Age-related analysis showed elevated suicide risk in patients aged <40 or 60–79 years, with the highest risk in those under 40 years (SMR = 2.15, 95% CI: 1.60–2.88). Patients aged 40–49 years showed reduced risk (SMR = 0.58, 95% CI: 0.41–0.82; Fig. 4; Supplementary Table S5) in the non-overlapping main analysis; however, this did not persist in the pooled analysis including all studies regardless of possible patient overlap (SMR = 0.79, 95% CI: 0.56–1.12; Supplementary Figure S3). No significant association was observed for those between 50 and 59 years (SMR = 1.21, 95% CI: 0.76–1.92) and those 80 years and older (SMR = 0.96, 95% CI: 0.71–1.30). Elevated suicide risks were consistent across regions, with SMRs ranging from 1.34 (95% CI: 1.11–1.62) in North America^53^ to 1.52 (95% CI: 1.24–1.87) in Eastern Europe.^14^^,^^16^ Rectal cancer patients had a slightly (but not significantly) higher risk (SMR = 1.67, 95% CI: 1.25–2.23) than those with colon cancer (SMR = 1.53, 95% CI: 1.27–1.83). Patients with metastatic disease faced a significantly higher risk (SMR = 3.63, 95% CI: 2.99–4.41) compared to non-metastatic cases (SMR = 1.38, 95% CI: 1.22–1.57). Suicide risk peaked within six months after diagnosis (SMR = 2.69, 95% CI: 1.29–5.61) and declined over time (e.g., 10 years after diagnosis: SMR = 0.81, 95% CI: 0.23–2.83). Recruitment period only had minimal impact, with SMRs ranging from 1.36 (95% CI: 1.24–1.48) before 1980 to 1.49 (95% CI: 1.29–1.71) between 2000 and 2010. To determine whether more recent improvements in therapy and screening methods influenced the risk of suicide in patients with colorectal cancer, we conducted an additional meta-analysis of studies that only included patients recruited after 2000 (SMR = 1.63, 95% CI: 1.41–1.90). For further information on subgroup analyses, see Fig. 4 and Supplementary Table S5.

Repeating the subgroup analyses with all 34 SMR studies yielded no material changes in suicide risk estimates, except for age-group findings noted above (see Supplementary Figure S3).

### Complementary analyses

Studies reporting HRs showed an elevated suicide risk (HR = 1.75, 95% CI: 1.55–1.97), which remained similar when including studies with potential patient overlap (HR = 1.70, 95% CI: 1.54–1.87; Supplementary Figure S4). For publication bias assessment, see Supplementary Figure S5. OR studies also indicated increased risk (OR = 2.56, 95% CI: 2.02–3.23; Supplementary Figure S6).

## Discussion

This study systematically reviewed the risk of suicide and suicidal ideation in patients with colorectal cancer, aiming to identify high-risk subgroups by gender, age, region, diagnosis, disease extent, time since diagnosis, and recruitment period. Pooled results showed a 40% higher suicide risk for patients with colorectal cancer compared to the general population, with a particularly high risk in patients with metastatic disease, those under 40 years, and within six months of diagnosis.

Compared to other cancer sites, colorectal cancer generally shows a lower suicide risk than lung, pancreatic, or head and neck cancers.^5^^,^^83^, ^84^, ^85^ One plausible explanation for this is a better prognosis for colorectal cancer: for example, 5-year survival for lung cancer is around 28% vs. 65% for colorectal cancer.^86^

However, comparable within-site patterns emerge across various malignancies: patients with advanced stages have higher suicide risk in lung, breast, and pancreatic cancer compared to earlier stages,^83^^,^^84^^,^^87^ and shorter time since diagnosis corresponds to greater risk, particularly in lung, head and neck, and pancreatic cancer.^83^, ^84^, ^85^

Suicidal ideation was analysed as a secondary outcome. Although no included study found a significantly increased risk of suicidal ideation among patients with colorectal cancer, there were indications of a potential upward trend when compared to cancer-free individuals.^29^ It is also possible that patients may be reluctant to disclose suicidal ideation due to factors such as stigmatisation, fear of judgment, or desire to protect loved ones.^88^ Moreover, suicidal ideation is inherently difficult to assess; often underreported, misclassified, or only indirectly addressed as a symptom of mental disorders.^89^ Given the limited number of studies directly comparing patients with colorectal cancer to the general population, such trends remain inconclusive. Nonetheless, the apparent disconnect between observed suicide risk and reported ideation underscores the need for more nuanced and sensitive screening approaches to better identify individuals at risk.

While men generally face a higher suicide risk,^2^ we found no significant difference between male and female patients with colorectal cancer, with both showing elevated risks. This aligns with prior meta-analyses across all cancer types, which report comparable risks between genders.^5^^,^^90^

Regarding age, several studies reported an increased suicide risk for younger patients with colorectal cancer.^13^^,^^21^^,^^47^^,^^53^^,^^54^ Consistent with these findings, patients aged 14–40 years showed the highest suicidality across all age groups. While a cancer diagnosis is challenging at any age, colorectal cancer in younger adults raises unique concerns. This life stage often involves major personal and professional transitions, which may amplify the psychological burden of the disease.^91^ During these transitions, young individuals may struggle with body image, sexual identity, gaining independence, and making life decisions,^92^ all of which can be further disrupted by the disease, resulting in financial strain, relationship adjustments, and the distressing confrontation with potential premature death.^93^ Treatment-related complications like urinary and sexual dysfunction can be particularly distressing for younger patients, as sexuality and fertility are often still being established.^94^ The apparent reduction in risk among patients aged 40-49 years in the non-overlapping analysis likely reflects reliance on a single contributing study (Pham et al.).^13^ Given the limited evidence in younger patients and the risk of duplicate participants across studies, we restricted inclusion to this study, selected for its methodological quality and for age strata consistent with our pre-specified categories. In a sensitivity analysis pooling all 34 SMR studies irrespective of potential overlap, the association for ages 40-49 was not significant. Accordingly, we interpret the primary finding with caution. If a true effect exists, plausible contributors include greater social integration, parental roles, fewer comorbidities, better functional status, potential differences in treatment response and established coping mechanisms. Additional studies focusing on younger patients are needed to increase power and permit stronger conclusions.

Our subgroup analysis of five regions (Western and Eastern Europe, Scandinavia, North America, and Asia) revealed elevated suicide risks across all, with no region showing significantly higher risk than the others. Among these five regions, Eastern Europe had the highest suicide risk in the general population, with a reported mortality rate of 19.2 per 100,000 in 2021.^1^ Although that study examined the general population, its regional trend parallels our findings among patients with colorectal cancer, with Eastern Europe exhibiting the highest suicide risk. High alcohol consumption, particularly strong spirits, might drive this trend.^95^ While mental health care is already improving in this region, further efforts are needed to ease the psychological burden.^96^ Contrary to Heinrich et al.,^5^ who found the highest suicidality among U.*S*. cancer patients, our study showed the lowest suicide risk in U.S. colorectal cancer patients. This may be due to the exclusion of 14 studies based on the Surveillance, Epidemiology, and End Results (SEER) database for potential patient overlap, leaving only one study for the regional sub-analysis.^53^ We selected Liu et al.^53^ for its high NOS rating, large sample over a long period, and detailed subgroup analyses. In contrast, Heinrich et al.^5^ included seven U.S. studies with non-overlapping data. When all eligible studies were included, the previously observed lower suicide risk among U.S. patients diminished, with regional rankings shifting in a way that supports our reasoning.

Rectal cancer patients showed a slightly higher, though not significant, suicide risk compared to those with colon cancer. This may reflect the greater burden from stomas, sexual dysfunction, and incontinence in rectal cancer patients.^97^ Stomas, for example, are indicated for locally advanced rectal cancer and might impact quality of life through body image or physical issues,^98^ potentially increasing mental burden. Therefore, our findings indicate that rectal cancer patients require frequent suicide screening and increased psycho-oncological care. Given that rectal cancer accounts for fewer new cases annually, the additional resources required are unlikely to place a significant burden on healthcare systems.^6^

Like Heinrich et al.,^5^ our analysis found significantly higher suicide risk in patients with advanced disease. Colorectal cancer treatment varies by type and stage. Early detection improves outcomes, while metastatic cases often need systemic therapies with potentially severe side effects.^99^ Fear of these, longer treatments, and the overall feeling of helplessness can increase suicidal thoughts,^100^ with some patients seeing suicide as a way to regain control.^5^

We found no significant difference in suicide risk between patients diagnosed in earlier vs. recent decades. This may seem surprising, as recent advances in colorectal cancer therapy, including expanded therapy options and thereby improved outcomes, might be expected to reduce suicide risk.^101^ Despite these improvements, a cancer diagnosis remains a major psychological burden.^3^ Advances in therapy, like new surgical techniques, may not ease key issues, such as living with a stoma or sexual dysfunction.^94^^,^^98^ However, to further evaluate the potential impact of improved cancer therapies as well as changes in suicide screening procedures, we conducted an additional analysis only including studies with start of recruitment after 2000. We chose 2000 as cutoff since, in the early 2000s, significant changes occurred in both therapeutic strategies and suicide screening procedures. In terms of therapy, novel cytotoxic agents, such as oxaliplatin and irinotecan, and monoclonal antibody therapies targeting EGF and VEGF receptors, have been introduced.^102^, ^103^, ^104^ Regarding screening procedures, the Distress Thermometer was implemented as the first systematic tool for assessing distress.^105^ However, results were similar to the main analysis, indicating that recruitment decade and associated changes in treatment or screening did not materially alter suicide risk.

Time since diagnosis is a key suicide risk factor, with patients facing over 2.5 times higher risk in the first six months. Beyond five years post-diagnosis, suicide risk approaches that of the general population, likely reflecting patients’ psychological adjustment, coping strategies, and access to psycho-oncological care over time.^83^ Furthermore, disease-related pain and therapy side effects tend to diminish over time.^106^ This, along with less frequent follow-up doctor visits and screenings, may explain the decreasing suicide risk over time, a trend also seen in other meta-analyses.^5^^,^^83^

Prior research identifies psychiatric morbidity as key risk factor for suicide.^107^, ^108^, ^109^ However, among the studies included in our review, only three accounted for psychiatric comorbidities,^4^^,^^12^^,^^67^ and results were heterogeneous: Sun et al.^12^ reported a nearly fourfold higher suicide risk among colorectal cancer patients with pre-existing depression, whereas Choi et al.^4^ found no significant association for mental disorders. Differences in the choice of reference groups precluded a meta-analysis of this dimension. Standardised definitions of mental comorbidities and comparators, with careful adjustment for confounding, are needed in future studies to clarify the magnitude of the effect.

Care teams for patients with colorectal cancer should prioritise timely recognition of emotional distress that can evolve into suicidal thoughts, particularly in the identified high-risk groups. Given the time pressure of routine oncology care, standardised brief screening is a practical entry point. Tools such as the Patient Health Questionnaire-9,^110^ the Distress Thermometer,^111^ or the Columbia Suicide Severity Rating Scale^112^ are widely used and can be integrated into waiting-room questionnaires. When resources are limited, a single-question screen may serve as a rapid preliminary flag for suicide risk and can be used as a basis for transfer to psycho-oncological or psychiatric care. The effectiveness of a single-question-based screening in cancer patients has been assessed in a Chinese study using the simple, yet tactful question, ‘During the last month, how much time have you felt meaningless in living?’ which showed promising potential as a preliminary screening tool for suicide risk.^113^ A single, straightforward question minimises barriers for caregivers and patients and fits seamlessly into most clinical settings. Beyond screening, proactive and continuous caregiving within the clinical setting plays a key role in preventing suicide among cancer patients. Screening should therefore be done at diagnosis and at key clinical transitions such as treatment initiation, completion, or recurrence, and whenever clinicians, patients, or support persons suspect emotional distress. Positive screening then must be followed with same-day referrals to psycho-oncology for persistent distress or urgent psychiatric evaluation when imminent risk is identified. At the service level, this might require increased efforts to train specialised psycho-oncological professionals.^114^

This is the first meta-analysis on suicide risk in patients with colorectal cancer, featuring a comprehensive search of six databases over an extended period, providing broad data coverage across Europe, Asia, and North America. We meta-analysed studies by SMRs, HRs, and ORs separately to ensure precise risk assessment and strengthen reliability, as all showed consistent suicide risk trends in patients with colorectal cancer. By including results on suicidal ideation, we offer a comprehensive view of the entire suicide process, beyond just fatal outcomes. We ensured high scientific and statistical quality by following relevant guidelines and by excluding studies that incorporate similar data sources, thereby minimising potential biases related to patient overlap. However, some limitations should be mentioned: First of all, fifteen studies analysed data from the SEER database, which resulted in only Liu et al.^53^ being included in the main analysis. This exclusion resulted in a loss of information but effectively prevented double-counting of patients and overrepresentation of certain databases.^42^ The same issue affected individual subgroup analyses, as excluding studies with potential patient overlap limited the number of studies available and made it difficult to draw valid conclusions for some groups.

A further limitation is cross-study heterogeneity. Included studies differed in methodological quality, effect measures, classification systems, and outcome ascertained (e.g., national registries vs. hospital records); study populations ranged from small regional cohorts to nationwide datasets; and data were collected across heterogeneous time periods with evolving treatment practices. We mitigated these issues by harmonising exposure and outcome definitions, pooling effect measures in separate analyses, and conducting random-effects, subgroup, and sensitivity analyses restricted to lower-heterogeneity sets. Nonetheless, some residual heterogeneity and misclassifications are possible. Future studies should focus on the impact of colorectal cancer on suicidality in low-income countries and the influence of additional risk factors (e.g., therapy, mental health, or ethnicity), which we were unable to investigate due to insufficient data.

In conclusion, compared to the general population, the risk of committing suicide among patients with colorectal cancer was increased by 40%. The highest suicide risk was observed in patients with metastatic cancer, those recently diagnosed, and younger patients. At the same time, suicidal ideation did not differ between patients with colorectal cancer and respective reference groups. This discrepancy may reflect limitations in current assessment methods and highlights the importance of refining screening tools. Our findings can raise caregiver awareness, enabling targeted suicide risk screening and psycho-oncological care for high-risk subgroups, thereby enhancing prevention and optimising resource allocation.

## Contributors

SB and PB have access to and verify the underlying study data.

Conceptualization, data curation, formal analysis: SB, PB; Supervision: PB, CSB, ML; writing - original draft: SB, PB; writing - review & editing: CSB, ML.

All authors had final responsibility for the decision to submit for publication.

## Data sharing statement

The data that support the findings of this study are available from the corresponding author (Patricia.Bohmann@ur.de) upon reasonable request.

## Declaration of interests

All authors declare no competing interests.
