# Supplementary material for: Suicide mortality and suicidal ideation among patients with colorectal cancer: a systematic review and meta-analysis
Source: eClinicalMedicine. 2025 Nov 28;90:103670. doi: 10.1016/j.eclinm.2025.103670 (PMC12704310; doi:10.1016/j.eclinm.2025.103670)
Supplement: Supplementary Material [file mmc1.pdf]

Suicide mortality and suicidal ideation among patients with colorectal cancer: a systematic review and meta-analysis

Supplementary Information

Supplementary Tables

Table S1: Search strategy and terms
- **(A)** PubMed (MEDLINE)
- **(B)** EMBASE (Ovid)
- **(C)** PsycINFO
- **(D)** CINAHL
- **(E)** Web of Science
- **(F)** Google Scholar

Table S2: International Classification of Diseases 10th Revision - Codes regarding colorectal cancer diagnosis

Table S3: Assessment of study quality using New Castle Ottawa Scale
- **(A)** Studies providing SMRs
- **(B)** Studies providing HRs
- **(C)** Studies providing ORs
- **(D)** Studies on suicidal ideation (all ORs)

Table S4: Characteristics of studies meeting inclusion criteria for systematic review

Table S5: Subgroup Analyses: Results of meta-regression tests

Supplementary Figures

Figure S1: Forest plot sensitivity analysis of SMR studies

Figure S2: Funnel plots for analyses of SMR studies
- **(A)** Nine non-overlapping studies (main analysis)
- **(B)** All 34 studies (sensitivity analysis)

Figure S3: Forest plot subgroup analysis, including all 34 SMR studies

Figure S4: Forest plots meta-analyses of HR-studies
- **(A)** Six non-overlapping studies
- **(B)** All eight studies

Figure S5: Funnel plot meta-analysis of HR studies
- **(A)** Six non-overlapping studies
- **(B)** All eight studies

Figure S6: Forest plot meta-analysis of OR studies

Table S1 A-F. Search strategy and terms.

Our search strategy included three key concepts connected by the Boolean operator “AND”: suicidal behavior, colorectal cancer, and epidemiologic studies. To maximize sensitivity, we searched for MeSH terms and keywords within titles and abstracts. To build the suicidal behavior concept, we consulted three previous Cochrane reviews.[1–3] To develop the colorectal cancer search concept, we systematically reviewed PubMed for relevant MeSH terms and keywords used in prior studies on colorectal cancer. We limited our search to epidemiologic studies by using the third concept, which was based on a study by Larney et al..[4] We ensured compliance with the recommendations of the PRESS[5] guideline. There was no restriction regarding the publication date. The search terms were initially developed for PubMed and subsequently adapted for other databases. Literature search was performed according to a linear search algorithm. In a first step, the single sets were created. Subsequently, the intersection of the sets 1, 2, and 3 was done. Date of last search of all databases was May 31, 2025.

(A) Pubmed (MEDLINE):

#	Search	Remarks	Records
1	((((“Colorectal Neoplasms”[Mesh]) OR “Colorectal Neoplasms, Hereditary Nonpolyposis”[Mesh]) OR “Colitis-Associated Neoplasms”[Mesh]) OR “Lynch Syndrome II”[Mesh]) OR (“colorectal cancer*”[tiab] OR cancer[ti] OR “colorectal carcinoma*”[tiab] OR “colon cancer*”[tiab] OR “colon carcinoma*”[tiab] OR “rectal cancer*”[tiab] OR “rectal carcinoma*”[tiab] OR “bowel cancer*”[tiab] OR “bowel neoplas*”[tiab] OR “colorectal tumor*”[tiab] OR “colorectal tumour*”[tiab] OR “colon tumor*”[tiab] OR “colon tumour*”[tiab] OR “rectal tumor*”[tiab] OR “rectal tumour*”[tiab] OR “bowel tumor*”[tiab] OR “bowel tumour*”[tiab] OR “colorectal cancer* pain”[tiab] OR “colorectal cancer* screening”[tiab] OR “early-onset colorectal cancer*”[tiab])	colorectal cancer with overall cancer in the title	1,542,408
2	((suicide[mesh] OR euthanasia[mesh]) OR (suicid*[tiab] OR "suicid* process"[tiab] OR suicidality[tiab] OR "suicid* event"[tiab] OR "suicid* attempt*"[tiab] OR "attempted suicid*"[tiab] OR "assist* death"[tiab] OR euthanasia[tiab] OR "medic* assist* in dying"[tiab] OR "aid in dying"[tiab]) OR ("Suicidal Ideation"[Mesh] OR ("suicid* behavior"[tiab] OR "suicid* behaviour"[tiab] OR "acute suicid* affective disturbance"[tiab] OR "suicid* ideation"[tiab] OR "suicid* thoughts"[tiab] OR "suicid* risk"[tiab])))	suicide, suicidal ideation, assisted death	153,006
3	((prevalence[tiab] OR incidence[tiab] OR epidemiol*[tiab] OR survey[tiab] OR cohort[tiab] OR surveillance[tiab] OR “long-term”[tiab] OR	epidemiologic studies	10,602,858

	longterm[tiab] OR “follow-up”[tiab] OR followup[tiab] OR case control[tiab] OR cross-sectional[tiab] OR crosssectional[tiab]) OR (epidemiologic methods[mesh] OR epidemiologic studies[mesh] OR sentinel surveillance[mesh] OR cohort studies[mesh] OR cross-sectional studies[mesh] OR longitudinal studies[mesh] OR follow-up studies[mesh] OR prospective studies[mesh]))		
4	1 AND 2 AND 3		1,113

(B) EMBASE (Ovid)

#	Search	Remarks	Records
1	exp colon cancer/ or exp colon tumor/ or exp rectum carcinoma/ or exp colorectal cancer/ or exp colorectal tumor/ or exp colon carcinoma/ or exp rectum cancer/ or exp rectum tumor/ or (colorectal cancer* or colorectal carcinoma* or colon cancer* or colon carcinoma* or rectal cancer* or rectal carcinoma* or bowel cancer* or bowel neoplasm* or colorectal tumor* or colorectal tumour* or colon tumor* or colon tumour* or rectal tumor* or rectal tumour* or bowel tumor* or bowel tumour* or colorectal cancer* pain or colorectal cancer* screening or early-onset colorectal cancer*).ti,ab,kw. or cancer.ti.	colorectal cancer with overall cancer in the title	2,190,735
2	exp suicide/ or exp suicidal ideation/ or exp suicidal behavior/ or exp suicide attempt/ or exp active euthanasia/ or exp voluntary euthanasia/ or exp euthanasia/ or exp assisted suicide/ or (suicid* or suicid* process or suicidality or suicid* event or suicid* attempt* or attempted suicid* or assist* death or euthanasia or medical assist* in dying or aid in dying).ti,ab,kw. or exp suicidal ideation/ or (suicid* behavior or suicid* behaviour or acute suicid* affective disturbance or suicid* ideation or suicid* thoughts or suicid* risk).ti,ab,kw.	suicide, suicidal ideation, assisted death	212,911
3	(prevalence or incidence or epidemiol* or survey or cohort or surveillance or “long-term” or longterm or “follow-up” or followup or case control or cross-sectional or crosssectional).ti,ab,kw. or exp epidemiology/ or exp epidemiological data/ or observational study/ or cohort analysis/ or longitudinal study/ or exp case control study/	epidemiologic studies	11,376,515
4	1 AND 2 AND 3		1,899
5	Limit 4 to (human and (english or german) and (article or article in press or conference paper or		1,151

	data paper or erratum or "preprint (unpublished, non-peer reviewed)" or "review" or short survey))		

(C) PsycINFO

#	Search	Remarks	Records
1	"colorectal cancer*" OR "colorectal carcinom*" OR "colon cancer*" OR "colon carcinom*" OR "rect* cancer*" OR "rect* carcinom*" OR "bowel cancer*" OR "bowel neoplasm*" OR "colorectal tumor*" OR "colorectal tumour*" OR "colon tumor*" OR "colon tumour*" OR "rectal tumor*" OR "rectal tumour" OR "bowel tumor" OR "bowel tumour" OR "colorectal cancer screening" OR TI cancer	colorectal cancer with overall cancer in the title	47,321
2	((DE "Suicide" OR DE "Suicidality" OR DE "Suicidal Behavior" OR DE "Attempted Suicide" OR DE "Suicidal Ideation" OR DE "Suicide Prevention" OR DE "Assisted Suicide" OR DE "Euthanasia") OR (MM "Euthanasia")) OR (suicid* OR "suicid* ideation" OR "suicid* behavior" OR "suicid* behaviour" OR "suicid* attempt" OR euthanasia OR "assisted suicide" OR "suicid* process" OR suicidality OR "suicid* event" OR "attempt* suicid*" OR "assist* death" OR "medic* assist* in dying" OR "aid in dying" OR "suicid* thoughts" OR "suicid* risk")	suicide, suicidal ideation, assisted death	92,973
3	DE "Epidemiology" OR (prevalence OR incidence OR epidemiol* OR survey OR cohort OR surveillance OR "long-term" OR longterm OR "follow-up" OR followup OR "case control" OR cross-sectional OR crosssectional OR "epidemiology" OR "epidemiological data" OR "observational study" OR "cohort analysis" OR "longitudinal study" OR "case control study")	epidemiologic studies	1,247,857
4	1 AND 2 AND 3		250

(D) CINAHL

#	Search	Remarks	Records
1	((MH "Colorectal Neoplasms, Hereditary Nonpolyposis+") OR (MH "Colorectal Neoplasms+") OR (MH "Colitis-Associated Neoplasms")) OR ("colorectal cancer*" OR "colorectal carcinom*" OR "colon cancer*" OR "colon carcinom*" OR "rect* cancer*" OR "rect* carcinom*" OR "bowel cancer*" OR "bowel neoplasm*" OR "colorectal tumor*" OR "colorectal tumour*" OR "colon tumor*" OR "colon tumour*" OR "rectal tumor*" OR "rectal	colorectal cancer with overall cancer in the title	411,602

	tumour" OR "bowel tumor" OR "bowel tumour" OR "colorectal cancer screening" OR TI cancer)		
2	((MH "Suicide+") OR (MH "Suicide, Assisted") OR (MH "Suicide Prevention") OR (MH "Suicide, Attempted") OR (MH "Suicide Risk (Saba CCC)") OR (MH "Suicide Prevention (Iowa NIC)") OR (MH "Suicide Self-Restraint (Iowa NOC)") OR (MH "Suicidal Ideation")) OR (suicid* OR "suicid* ideation" OR "suicid* behavior" OR "suicid* behaviour" OR "suicid* attempt" OR euthanasia OR "assisted suicide" OR "suicid* process" OR suicidality OR "suicid* event" OR "attempt* suicid*" OR "assist* death" OR "medic* assist* in dying" OR "aid in dying" OR "suicid* thoughts" OR "suicid* risk"))	suicide, suicidal ideation, assisted death	63,161
3	((MH "Epidemiological Research+") OR (MH "Epidemiology+")) OR (prevalence OR incidence OR epidemiol* OR survey OR cohort OR surveillance OR "long-term" OR longterm OR "follow-up" OR followup OR "case control" OR cross-sectional OR crosssectional OR "epidemiology" OR "epidemiological data" OR "observational study" OR "cohort analysis" OR "longitudinal study" OR "case control study")	epidemiologic studies	2,293,358
4	1 AND 2 AND 3		425

(E) Web of Science

#	Search	Remarks	Records
1	(TS=("colorectal neoplasms" OR "colitis-associated neoplasms" OR "Lynch Syndrome II" OR "colorectal cancer*" OR "colorectal carcinoma*" OR "colon cancer*" OR "colon carcinoma*" OR "rectal cancer*" OR "rectal carcinoma*" OR "bowel cancer*" OR "bowel neoplasm*" OR "colorectal tumor*" OR "colorectal tumour*" OR "colon tumor*" OR "colon tumour*" OR "rectal tumor*" OR "rectal tumour*" OR "bowel tumor*" OR "bowel tumour*" OR "colorectal cancer* pain" OR "colorectal cancer* screening" OR "early-onset colorectal cancer*")) OR TI=(cancer)	colorectal cancer with overall cancer in the title	1,873,081
2	TS=("euthanasia" OR "suicid*" OR "suicid* process" OR "suicidality" OR "suicid* event" OR "suicid* attempt*" OR "attempted suicide" OR "assisted death" OR "medical assistance in dying" OR "aid in dying" OR "suicidal ideation" OR "suicidal behavior" OR "suicidal behaviour" OR	suicide, suicidal ideation, assisted death	140,062

	"acute suicidal affective disturbance" OR "suicidal thoughts" OR "suicidal risk")		
3	TS=("prevalence" OR "incidence" OR "epidemiol*" OR "survey" OR "cohort" OR "surveillance" OR "long-term" OR "longterm" OR "follow-up" OR "followup" OR "case control" OR "cross-sectional" OR "crosssectional" OR "sentinel surveillance" OR "cohort studies" OR "cross-sectional studies" OR "longitudinal studies" OR "follow-up studies" OR "prospective studies")	epidemiologic studies	6,953,100
4	1 AND 2 AND 3		928

(F) Google Scholar

#	Search	Remarks	Records
1	("colorectal cancer" OR "colon cancer" OR "rectal cancer" OR "colorectal neoplasm" OR “colorectal tumor” OR intitle: “cancer”) ("suicide" OR "suicidal ideation" OR “suicidality” OR "euthanasia" OR “assisted death” OR "aid in dying") ("epidemiologic study")	colorectal cancer AND suicide AND epidemiologic study	833

Table S2. International Classification of Diseases 10th Revision - Codes regarding colorectal cancer diagnosis.

ICD-10 Code	Description
**C18.-**	Malignant neoplasm of colon
**C18.0**	Malignant neoplasm of Caecum, Ileocaecal valve
**C18.1**	Malignant neoplasm of Appendix
**C18.2**	Malignant neoplasm of Ascending colon
**C18.3**	Malignant neoplasm of Hepatic flexure
**C18.4**	Malignant neoplasm of Transverse colon
**C18.5**	Malignant neoplasm of Splenic flexure
**C18.6**	Malignant neoplasm of Descending colon
**C18.7**	Malignant neoplasm of Sigmoid colon, Sigmoid (flexure);<br>Excl.: rectosigmoid junction (C19)
**C18.8**	Overlapping lesion of colon
**C18.9**	Malignant neoplasm of Colon, unspecified
**C19**	Malignant neoplasm of rectosigmoid junction;<br>Incl.:Colon with rectum, Rectosigmoid (colon)
**C20**	Malignant neoplasm of rectum;<br>Incl.: Rectal ampulla

Table S3 A-D. Assessment of study quality using New Castle Ottawa Scale.

(A) Studies providing SMRs:

First Author	Year	Title	PMID/DOI	S.B.	P.B.
**Allebeck**	1989	Increased suicide rate in cancer patients	2760653	7	7
**Allebeck**	1991	Suicides and suicide attempts in cancer patients	1780410	7	8
**Tanaka**	1999	Suicide Risk among Cancer Patients: Experience at One Medical Center in Japan, 1978–1994	10543251	7	7
**Hem**	2004	Suicide Risk in Cancer Patients From 1960 to 1997	15483032	8	8
**Misono**	2008	Incidence of Suicide in Persons With Cancer	18695257	8	8
**Ahn**	2010	Suicide Rates and Risk Factors among Korean Cancer Patients, 1993-2005	20696665	8	8
**Smailyte**	2013	Suicides among cancer patients in Lithuania: A population-based census-linked study	23809215	7	7
**Oberaigner**	2014	Increased suicide risk in cancer patients in Tyrol/Austria	25015541	8	8
**Ahn**	2015	Suicide in cancer patients within the first year of diagnosis	25336020	8	8
**Vyssoki**	2015	Suicide among 915,303 Austrian cancer patients: Who is at risk?	25661393	8	8
**Kaceniene**	2017	Increasing suicide risk among cancer patients in Lithuania from 1993 to 2012: a cancer registry-based study	28914692	8	8
**Rahouma**	2018	Lung cancer patients have the highest malignancy-associated suicide rate in USA: a population-based analysis	30174721	7	7
**Pham**	2018	Clinical and epidemiological factors associated with suicide in colorectal cancer	30027329	8	8
**Anderson**	2018	Suicide rates among patients with cancers of the digestive system	10.1002/pon.4827	8	8
**Henson**	2019	Risk of Suicide After Cancer Diagnosis in England	30476945	8	8
**Saad**	2019	covering 40 years of data	30613943	8	7
**Dulskas**	2019	Suicide risk among colorectal cancer patients in Lithuania	30617411	8	8
**Zaorsky**	2019	Suicide among cancer patients	30643135	8	8
**Oh**	2019	Causes of death among cancer patients in the era of cancer survivorship in Korea: Attention to the suicide and cardiovascular mortality	31960609	7	8
**Abdel-Rahman**	2019	Socioeconomic predictors of suicide risk among cancer patients in the United States: A population-based study	31536912	8	8

**Yang**	2021	Incidence of suicide among adolescent and young adult cancer patients	34663328	7	7
**Liu**	2022	Subsequent risk of suicide among 9,300,812 cancer survivors in US: A population-based cohort study covering 40 years of data	35198920	9	9
**Kurisu**	2022	Suicide, other externally caused injuries, and cardiovascular disease within 2 years after cancer diagnosis: A nationwide population-based study in Japan	35941747	8	8
**Su**	2022	Suicide and Cardiovascular Death Among Patients With Multiple Primary Cancers in the United States	10.3389/fcvm.2022.857194	8	8
**Hu**	2023	Suicide Risk Among Individuals Diagnosed With Cancer in the US, 2000-2016	36662522	8	8
**Potter**	2023	Incidence, Timing, and Factors Associated With Suicide Among Patients Undergoing Surgery for Cancer in the US	36633854	8	8
**Michalek**	2023	Suicide risk among adolescents and young adults after cancer diagnosis: analysis of 34 cancer groups from 2009 to 2019	36930435	8	8
**Michalek**	2023	Risk of suicide in patients with cancer aged 75 years or more – Follow-up of over 400,000 individuals	37348282	8	7
**Michalek**	2023	Suicide after a Diagnosis of Cancer: Follow-Up of 1.4 Million Individuals, 2009–2019	37686591	8	8
**Kinslow**	2023	Prognosis and risk of suicide after cancer diagnosis	38018695	7	7
**Chen**	2023	Causes of death among early-onset colorectal cancer population in the United States: a large population-based study	10.3389/fonc.2023.1094493	8	8
**Chen**	2020	Cause of death among patients with colorectal cancer: a population-based study in the United States	33289707	8	8
**Kitagawa**	2024	Suicide Risk Among Patients With Cancer by Sex in Japan: A Population-based Study	38462529	8	8
**Butare**	2025	Clinical and Demographic Factors Associated Suicide Risk in Patients With Colorectal Cancer	39798408	8	8

(B) Studies providing HRs:

First Author	Year	Title	PMID/DOI	S.B.	P.B.
**Riihimäki**	2012	Colorectal cancer patients: what do they die of?	10.1136/flgastro-2012-100141	8	8

**Sun**	2018	Risk of suicide attempts among colorectal cancer patients: A nationwide population-based matched cohort study	30225911	9	8
**Choi**	2019	Suicide risk after cancer diagnosis among older adults: A nationwide retrospective cohort study	31787493	8	8
**Klaasen**	2019	Cancer Diagnosis and Risk of Suicide After Accounting for Prediagnosis Psychiatric Care: A Matched-Cohort Study of Patients With Incident Solid-Organ Malignancies	31219606	8	8
**Choi**	2021	Suicide after cancer diagnosis in South Korea: a population-based cohort study	34475169	8	7
**Dent**	2022	Suicide risk following a new cancer diagnosis among Veterans in Veterans Health Administration care	10.1002/cam4.5146	7	7
**Forbes**	2024	Early, medium and long-term mental health in cancer survivors compared with cancer-free comparators: matched cohort study using linked UK electronic health records	39318789	9	9
**Larsson**	2024	Suicide after colorectal cancer—a national population-based study	38831481	9	8

(C) Studies providing ORs:

First Author	Year	Title	PMID/DOI	S.B.	P.B.
**Zhou**	2019	Suicide among cancer patients: adolescents and young adult (AYA) versus all-age patients	31930059	8	8
**Song**	2024	Suicide risk of chronic diseases and comorbidities: A Korean case-control study	10.1016/j.jad.2024.01.037	8	8

(D) Studies on suicidal ideation (all ORs):

First Author	Year	Title	PMID/DOI	S.B.	P.B.
**Kye**	2016	Suicidal ideation and suicidal attempts among adults with chronic diseases: A cross-sectional study	10.1016/j.comppsych.2016.12.001	9	8
**Schwinn**	2018	Prevalence of current suicidal thoughts and lifetime suicide attempts in individuals with cancer and other chronic diseases in Germany: Evidence for differential associations from a representative community cohort	39178957	7	8

**Walker**	2021	Suicidal Thoughts in Patients With Cancer and Comorbid Major Depression: Findings From a Depression Screening Program	34562640	8	8
**Hagezom**	2021	Magnitude and Associated Factors of Suicidal Ideation Among Cancer Patients at Ayder Comprehensive Specialized Hospital, Mekelle, Ethiopia, 2019: Cross-sectional Study	34103989	8	7
**Katayama**	2023	Suicidal Ideation Among Patients with Gastrointestinal Cancer	37061648	8	8

Table S4. Characteristics of studies meeting inclusion criteria for systematic review.

Author, Year	Country	Colorectal Cancer Patients	Suicides	Person-Years	Type of Risk estimate	Risk estimate (95% CI)
Allebeck et al., 1989	Sweden	n.a.	133	n.a.	SMR	2.10 (1.60-3.20)
Hem et al., 2004	Norway	n.a.	63	318,934	SMR	1.14 (0.75-1.68)
Vyssoki et al., 2015	Austria	n.a.	396	1,851,586	SMR	1.46 (1.32-1.61)
Dulskas et al., 2019	Lithuania	19,409	67	85,505.60	SMR	1.62 (1.27-2.06)
Oh et al., 2019	South Korea	334,320	673	n.a.	SMR	1.50 (1.30-1.69)
Henson et al., 2019	England	578,270	349	n.a.	SMR	1.28 (1.15-1.42)
Liu et al., 2022	USA	n.a.	721	n.a.	SMR	1.34 (1.24-1.44)
Michalek et al., 2023	Poland	178,267	134	n.a.	SMR	1.46 (1.22-1.73)
Kitagawa et al., 2024	Japan	93,805	195	413,682	SMR	1.36 (1.09-1.70)
Pham et al., 2018	USA	884,529	1,381	4,747,322	SMR	1.53 (1.13-1.33)
Chen et al., 2020	USA	834,510	1,360	n.a.	SMR	1.58 (1.50-1.67)
Hu et al., 2023	USA	1,611,161	1,992	n.a.	SMR	1.25 (1.20-1.31)
Kinslow et al., 2023	USA	634,028	n.a.	n.a.	SMR	2.79 (2.34-3.29)
Michalek et al., 2023	Poland	1,818	1	3,963	SMR	1.98 (0.05-11.50)
Kurisu et al., 2022	Japan	187,211	122	n.a.	SMR	1.84 (1.71-1.99)
Yang et al., 2021	USA	82,991	109	143,728.90	SMR	1.95 (1.43-2.65)
Abdel-Rahman et al., 2019	USA	315,795	439	n.a.	SMR	1.35 (1.22-1.48)
Saad et al., 2019	USA	453,774	131	n.a.	SMR	2.08 (1.74-2.47)
Rahouma et al., 2018	USA	n.a.	724	n.a.	SMR	1.41 (1.31-1.52)
Kaceniene et al., 2017	Lithuania	n.a.	87	n.a.	SMR	1.95 (1.58-2.41)
Ahn et al., 2015	South Korea	n.a.	54	68,563.50	SMR	1.14 (0.71-1.74)
Oberaigner et al., 2014	Austria	6,177	17	31,792	SMR	1.86 (1.08-2.97)
Smailyte et al., 2013	Lithuania	n.a.	32	25,192	SMR	2.73 (1.42-4.81)
Misono et al., 2008	USA	317,951	524	1,529,946	SMR	1.90 (1.74-2.07)
Tanaka et al., 1999	Japan	1,841	5	n.a.	SMR	2.37 (0.76-5.53)
Allebeck et al., 1991	Sweden	n.a.	15	n.a.	SMR	2.55 (1.05-5.20)
Allebeck et al., 1989	Sweden	n.a.	133	n.a.	SMR	2.10 (1.60-3.20)

Butare et al., 2025	USA	530,711	782	n.a.	SMR	1.36 (1.20-1.55)
Zaorsky et al., 2019	USA	n.a.	616	4,114.09	SMR	3.38 (3.12-3.66)
Chen et al., 2023	USA	36,013	n.a.	5,055,991	SMR	1.53 (1.21-1.90)
Anderson et al., 2018	USA	588,687	n.a.	1,984,376	SMR	1.39 (1.13-1.71)
Su et al., 2022	USA	64,427	92	249,792	SMR	2.05 (1.67-2.52)
Potter et al., 2023	USA	387,289	449	n.a.	SMR	1.28 (1.16-1.40)
Ahn et al., 2010	South Korea	108,940	285	322,869	SMR	1.84 (1.61-2.12)
Larsson et al., 2024	Sweden	64,855	96	n.a.	HR	1.71 (1.37-2.55)
Sun et al., 2018	Taiwan	96,470	150	359,399	HR	2.03 (1.60-2.56)
Forbes et al., 2024	United Kingdom	117,988	n.a.	n.a.	HR	1.87 (1.43-2.43)
Choi et al., 2021	South Korea	4,976	24	n.a.	HR	2.06 (1.45-3.25)
Choi et al., 2019	South Korea	9,570	15	n.a.	HR	1.56 (0.93-2.64)
Klaassen et al., 2019	Canada	119,241	n.a.	n.a.	HR	1.58 (1.27-1.96)
Riihimäki et al., 2012	Sweden	95,468	190	n.a.	HR	1.56 (1.34-1.82)
Dent et al., 2022	USA	15,363	26	n.a.	HR	1.48 (1.01-2.17)
Song et al., 2024	South Korea	n.a.	726	n.a.	OR	2.82 (2.61-3.05)
Zhou et al., 2019	USA	609,617	n.a.	n.a.	OR	2.21 (1.76-2.77)

Table S5. Subgroup Analyses: Results of meta-regression tests.

Category		SMR	95% CI	p-Value	q-Value
**Gender**				0.6608	0.6608
	Women (n=9)	1.42	1.23-1.63	/	
	Men (n=9)	1.48	1.23-1.76	0.6608	
**Age**				<0.0001	<0.0001
	< 40 years (n=2)	2.15	1.60-2.88	/	
	40-49 years (n=1)	0.58	0.41-0.82	< 0.0001	
	50-59 years (n=2)	1.21	0.76-1.92	0.0150	
	60-69 years (n=2)	1.71	1.17-2.49	0.2354	
	70-79 years (n=2)	1.77	1.23-2.55	0.3000	
	≥ 80 years (n=2)	0.96	0.71-1.30	< 0.0001	
**Region**				0.7735	0.7877
	Western Europe (n=2)	1.37	1.22-1.54	/	
	North America (n=1)	1.34	1.11-1.62	0.8262	
	Asia (n=2)	1.45	1.20-1.75	0.5322	
	Eastern Europe (n=2)	1.52	1.24-1.87	0.3230	
	Scandinavia (n=2)	1.51	1.10-2.09	0.5450	
**Diagnosis**				0.5395	0.5395
	Colon (n=4)	1.53	1.27-1.83	/	
	Rectum (n=3)	1.67	1.25-2.23	0.5395	
**Extent of disease**				<0.0001	<0.0001
	Non-metastatic (n=2)	1.38	1.22-1.57	/	
	Metastatic (n=2)	3.63	2.99-4.41	<0.0001	
**Time since diagnosis**				0.2304	0.2304
	≤5 years (n=8)	1.75	1.17-2.62	/	
	≤ 0.5 year (n=3)	2.69	1.29-5.61	0.2512	
	≤ 1 year (n=6)	2.25	1.20-4.21	0.4298	
	≤ 10 years (n=5)	1.35	0.72-2.53	0.4233	
	> 10 years (n=1)	0.81	0.23-2.83	0.2296	
**Start of Recruitment**				0.4669	0.5914
	before 1980 (n=3)	1.36	1.24-1.48	/	
	before 1990 (n=2)	1.44	1.25-1.65	0.5132	
	before 2000 (n=2)	1.34	1.16-1.54	0.8264	
	before 2010 (n=2)	1.49	1.29-1.71	0.2844	
	after 2010 (n=0)	n.a.	n.a	n.a.	

Reference groups were defined by the highest number of included studies and the overall literature preference. The first group of each category represents the reference group.

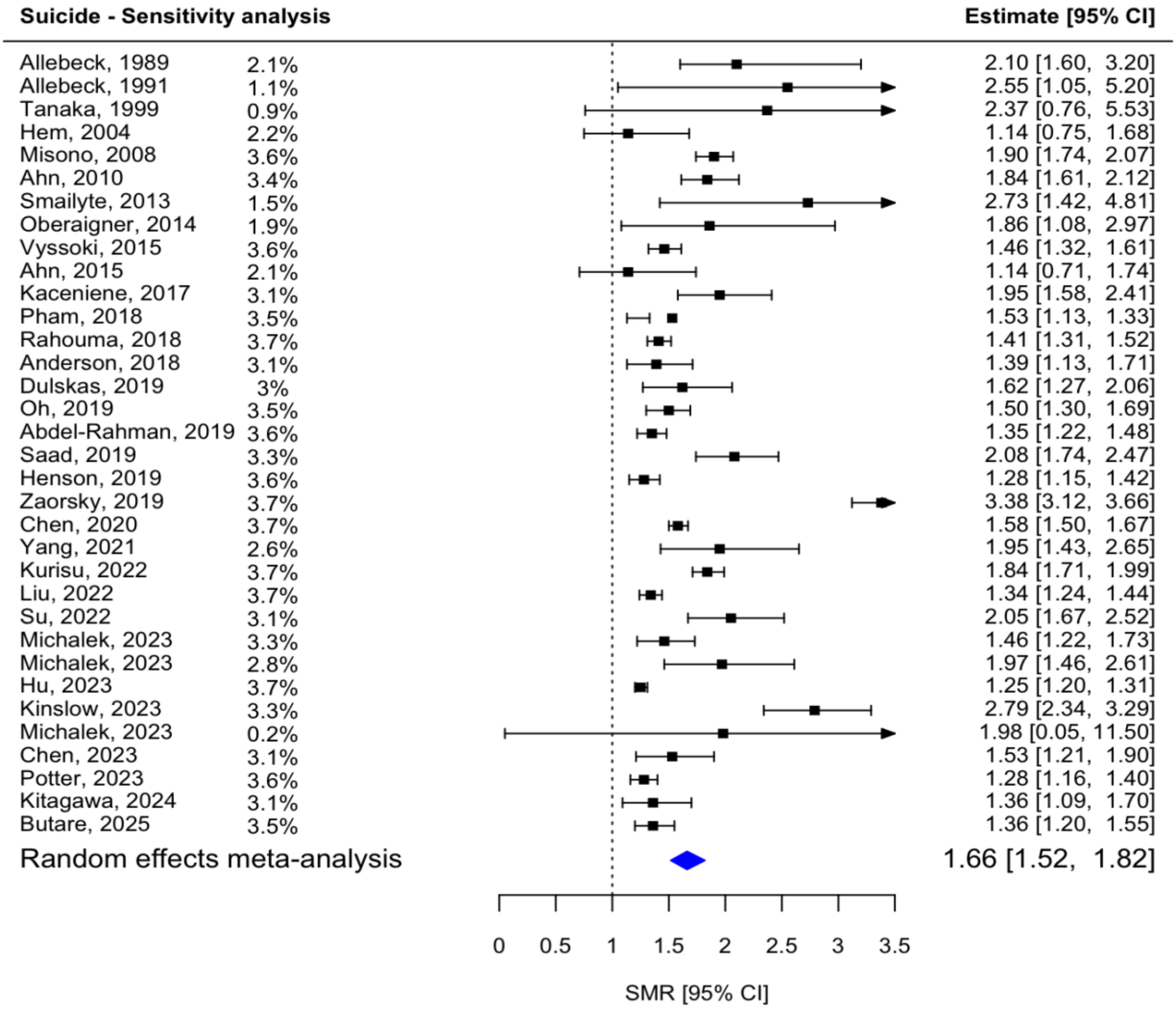

Supplementary Figure S1. Forest plot of random effects meta-analysis on suicide among colorectal cancer patients compared to the general population of all 34 studies providing SMR as RE (sensitivity analysis), I^2 = 93.99%, P-heterogeneity <0.001.

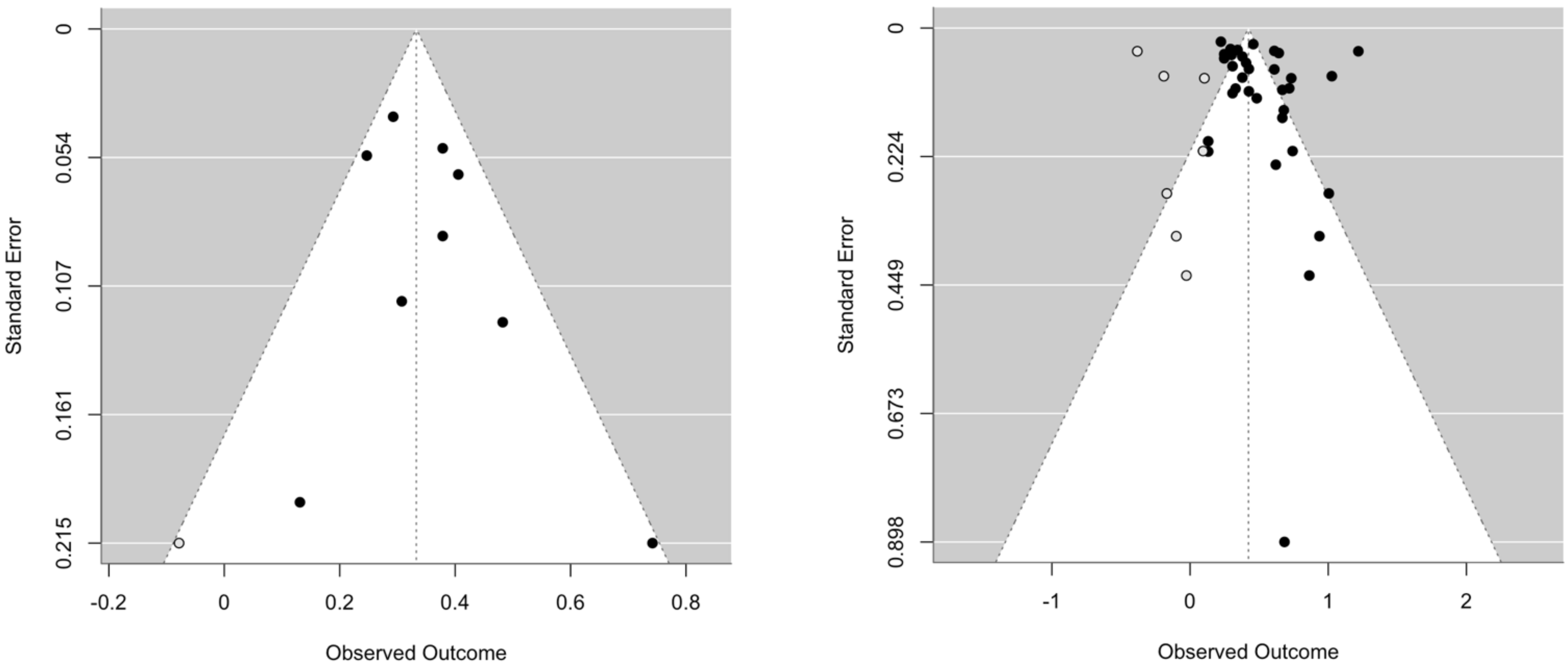

(A) **(B)**

Supplementary Figure S2. (A) Funnel plot for random effects meta-analysis including nine risk estimates (SMR) of suicide in colorectal cancer patients. p-value for Begg's test = 0.359; p-value for Egger's test = 0.209. **(B)** Funnel plot for random effects meta-analysis including all 34 risk estimates (SMR) of suicide in colorectal cancer patients. p-value for Begg's test = 0.204; p-value for Egger's test = 0.188.

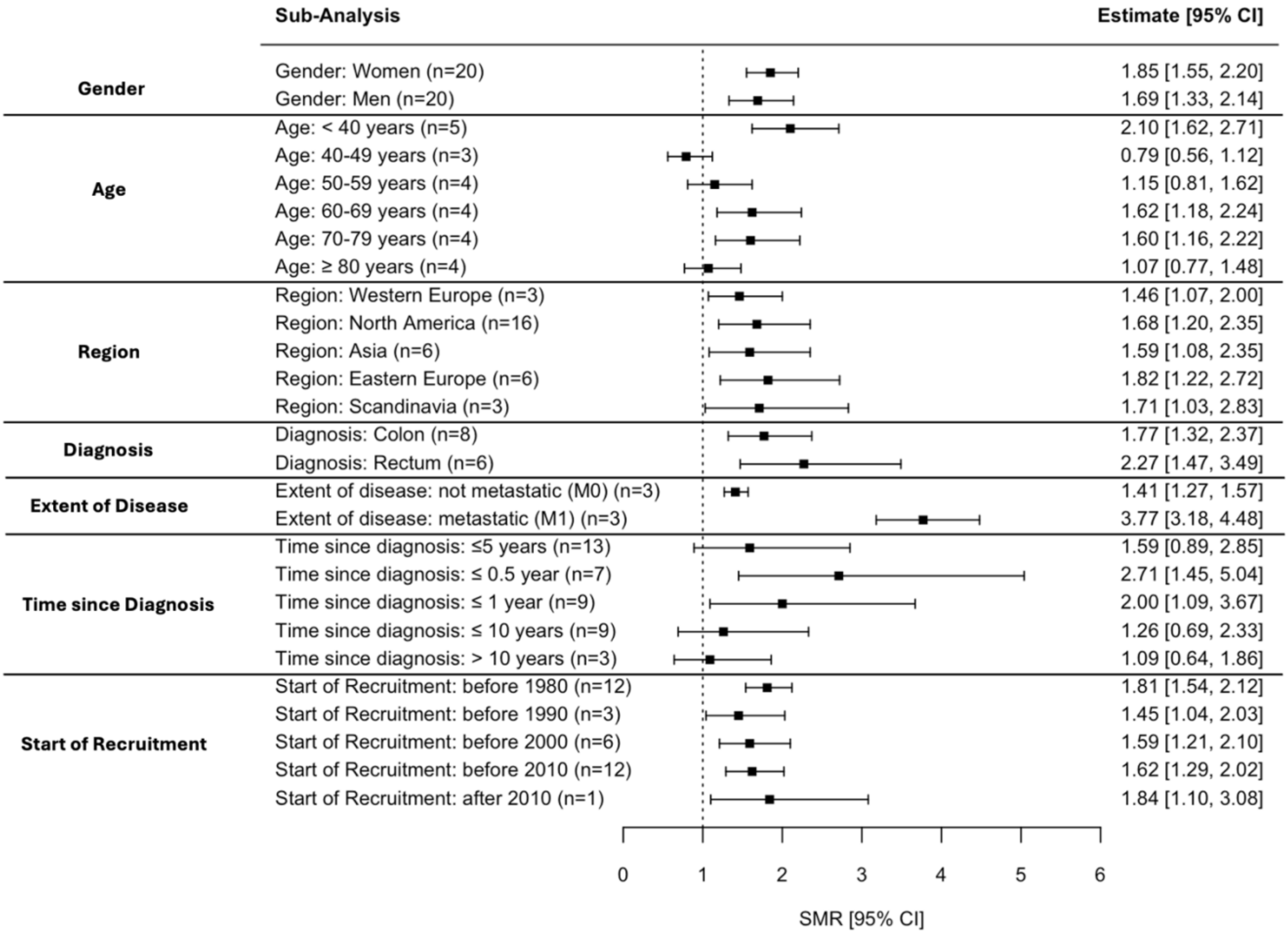

Supplementary Figure S3. Summary forest plot of subgroup analyses of suicide among colorectal cancer patients, including all 34 studies with potential patient overlap.

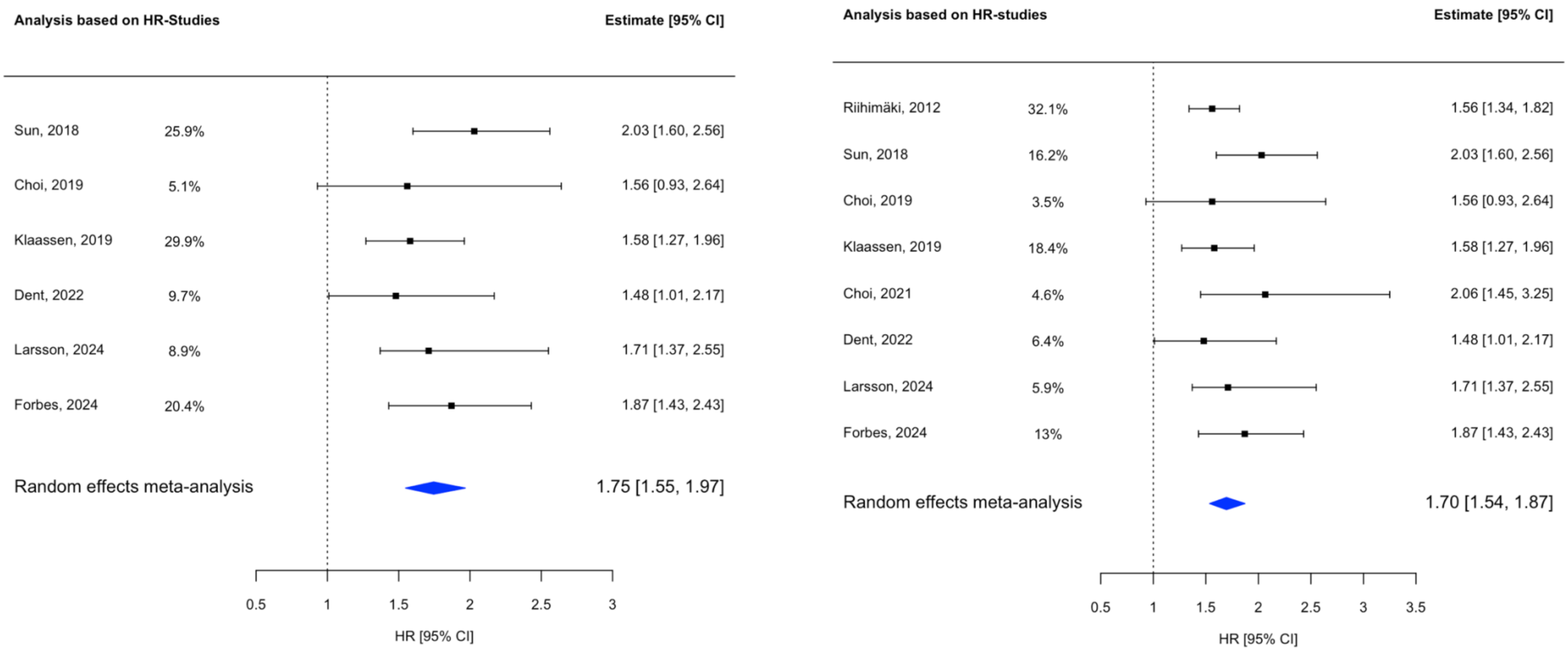

(A) **(B)**

Supplementary Figure S4. Forest plot of random effects meta-analysis of studies providing HRs on suicide among colorectal cancer patients compared to individuals without a colorectal cancer diagnosis. **(A)** Six non-overlapping studies (of suicide among colorectal cancer patients), I^2 = 1.53%, P-heterogeneity = 0.606. **(B)** All eight studies (of suicide among colorectal cancer patients), I^2 = 8.25%, P-heterogeneity 0.577.

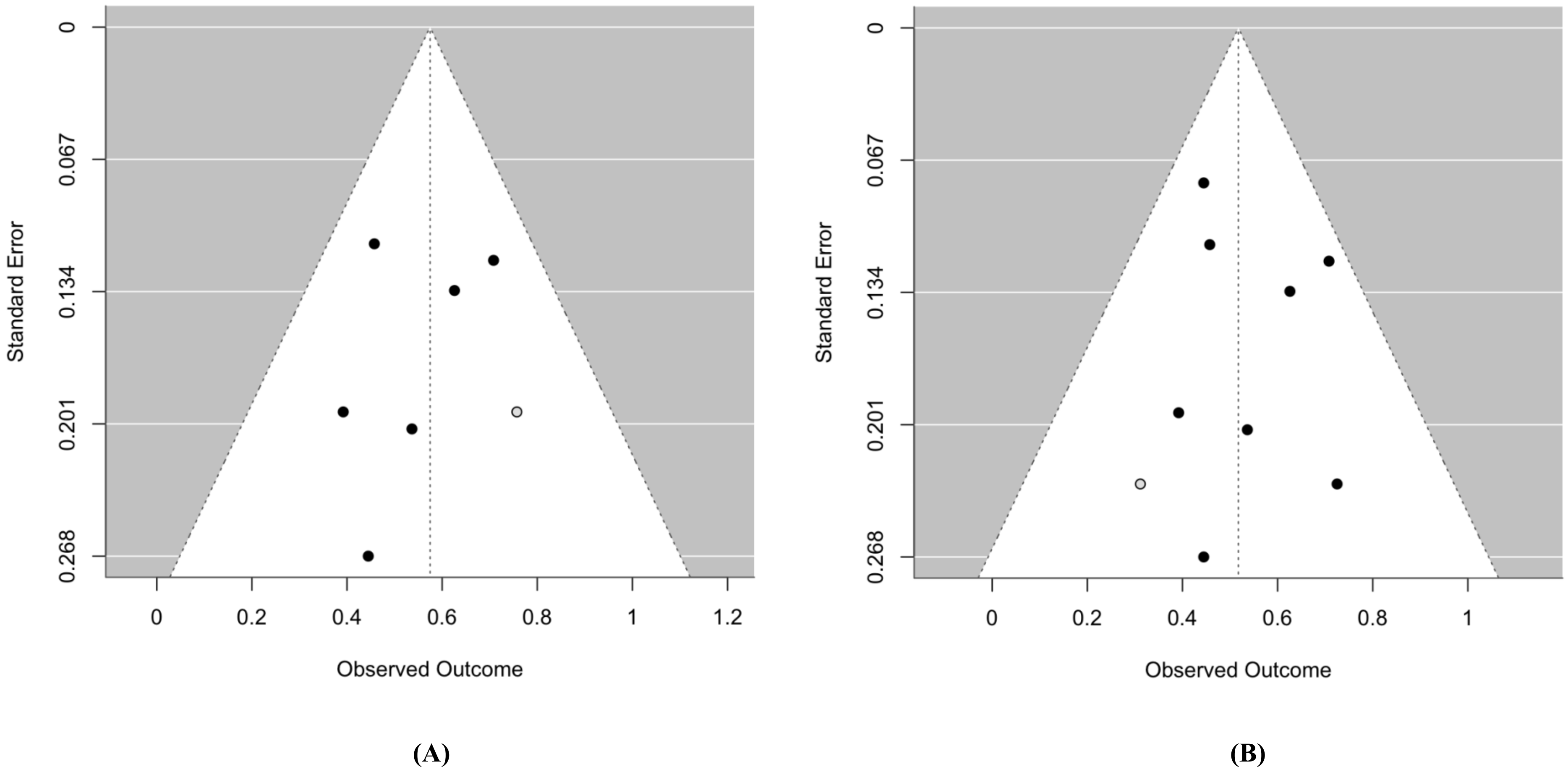

Supplementary Figure S5. (A) Funnel plot for random effects meta-analysis, including six risk estimates (HR) of suicide among colorectal cancer patients. p-value for Begg's test = 1.000; p-value for Egger's test = 0.505. **(B)** Funnel plot for random effects meta-analysis including all eight risk estimates (HR) of suicide among colorectal cancer patients. p-value for Begg's test = 0.548; p-value for Egger's test = 0.604.

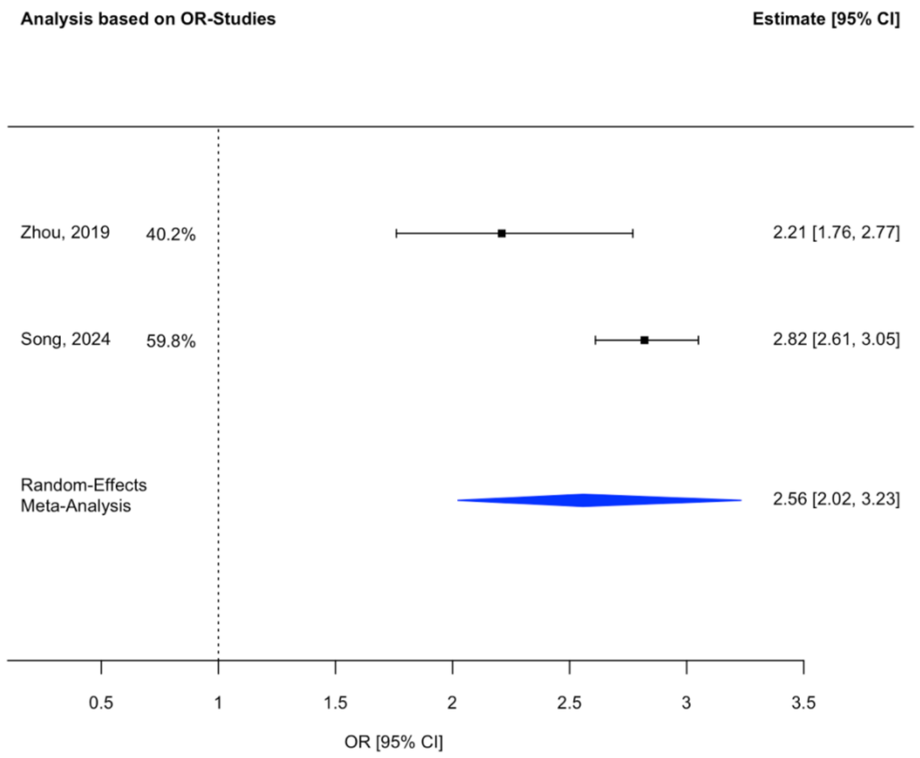

Supplementary Figure S6. Forest plot of random effects meta-analysis of two studies providing ORs on suicide among colorectal cancer patients compared to individuals without a colorectal cancer diagnosis, I^2 = 74.96%, P-heterogeneity 0.046.

References:

1. Okolie C, Hawton K, Lloyd K, Price SF, Dennis M, John A. Means restriction for the prevention of suicide on roads. *Cochrane Database Syst Rev*. 2020;9(9):CD013738. doi:10.1002/14651858.CD013738

2. Witt KG, Hetrick SE, Rajaram G, et al. Psychosocial interventions for self-harm in adults. *Cochrane Database Syst Rev*. 2021;2021(4):CD013668. doi:10.1002/14651858.CD013668.pub2

3. Saffari M, Pakpour AH, Mortazavi SF, Koenig HG. Psychometric characteristics of the Muslim Religiosity Scale in Iranian patients with cancer. *Palliat Support Care*. 2016;14(6):612-620. doi:10.1017/S1478951516000237

4. Larney S, Kopinski H, Beckwith CG, et al. Incidence and prevalence of hepatitis C in prisons and other closed settings: results of a systematic review and meta-analysis. *Hepatology*. 2013;58(4):1215-1224. doi:10.1002/hep.26387

5. McGowan J, Sampson M, Salzwedel DM, Cogo E, Foerster V, Lefebvre C. PRESS Peer Review of Electronic Search Strategies: 2015 Guideline Statement. *J Clin Epidemiol*. 2016;75:40-46. doi:10.1016/j.jclinepi.2016.01.021
